# The Road to Technical Proficiency in Cytoreductive Surgery for Peritoneal Carcinomatosis: Risk-Adjusted Cumulative Summation Analysis

**DOI:** 10.3389/fsurg.2022.877970

**Published:** 2022-05-18

**Authors:** Francesco Santullo, Carlo Abatini, Miriam Attalla El Halabieh, Federica Ferracci, Claudio Lodoli, Lorenzo Barberis, Francesco Giovinazzo, Andrea Di Giorgio, Fabio Pacelli

**Affiliations:** ^1^ Surgical Unit of Peritoneum and Retroperitoneum, Fondazione Policlinico Universitario A. Gemelli IRCCS, Rome, Italy; ^2^ General Surgery and Liver Transplant Unit, Fondazione Policlinico Universitario A. Gemelli IRCCS, Rome, Italy; ^3^ Dipartimento di Medicina e Chirurgia Traslazionale, Università Cattolica del Sacro Cuore, Rome, Italy

**Keywords:** peritoneal metastases, cytoreductive surgery (CRS), hyperthermic intraperitoneal chemotherapy (hipec), peritonectomy, learning curve, CUSUM, RA-CUSUM

## Abstract

**Background:**

Cytoreductive surgery (CRS) is a technically demanding procedure, and there is considerable debate about its safe application. This study investigated the learning curve for CRS and the clinical outcomes of consecutive patients treated by a single surgeon at a single institution.

**Methods:**

We collected 251 consecutive patients who underwent CRS for peritoneal metastases by a single surgeon at Fondazione Policlinico Universitario A. Gemelli IRCCS, between January 2016 and December 2020. The learning curve was estimated using the cumulative summation analysis (CUSUM) for operative time (OT). Risk-adjusted CUSUM (RA-CUSUM) charts were developed using a composite variable (surgical failure), defined as the occurrence of at least one of the following events: major postoperative complications (Clavien–Dindo grade ≥3), blood loss ≥500 mL, incomplete cytoreduction. Three learning phases were thus derived from the RA-CUSUM analysis, and were compared in terms of perioperative outcomes.

**Results:**

CUSUM-OT showed that the operation time improved significantly after the 161^th^ case. RA-CUSUM analysis allowed to break the CRS learning curve into three different phases: phase 1, “the learning phase” (cases 1–99), phase 2 “the experienced phase” (cases 100–188), and phase 3, “the mastership phase” (cases 189–251). The rate of major postoperative complications decreased significantly over the three phases (*p* = 0.019). Operative time decreased significantly as well (*p* = 0.031) and was significantly shorter in phase 3 with respect to the other two phases (phase 3 vs phase 2: 420 min vs 500 min, *p* = 0.017; phase 3 vs phase 1: 420 min vs 503 min, *p* = 0.021). Blood loss consistently decreased throughout the three phases (*p* = 0.001). The rate of incomplete cytoreduction was significantly lower in phase 3 than in phase 2 (4.8% vs 14.6%, *p* = 0.043).

**Conclusion:**

The CRS failure rate stabilized after the first 99 cases, and the complete surgical proficiency was achieved after 189 cases. A standardised and mentored learning model is a safer strategy to shorten the learning process, to reduce morbidity and mortality, to improve oncologic outcomes.

## Introduction

The peritoneal of gastrointestinal and gynaecological malignancies is usually associated with poor prognosis (1). Also, peritoneal carcinomatosis (PC) has been marginally affected by systemic chemotherapy and previously considered by the oncologists as an end-of-life condition, not amenable for surgery (2–4). Nowadays, the treatment of PC is radically changing thanks to a better understanding of tumours biology and their dissemination pattern. Therefore, PC is considered a form of loco-regional disease that could benefit from a multimodal approach combining aggressive surgery and chemotherapy (5).

Cytoreductive surgery (CRS) followed by hyperthermic intraperitoneal chemotherapy (HIPEC) is currently the standard of care in selected patients with PC from various abdominal malignancies (6, 7). CRS aims to remove every macroscopically visible tumour implant within the peritoneal cavity, and then a heated chemotherapy local infusion eradicates any residual microscopic disease (8). Therefore, CRS plus HIPEC results in a highly complex and lengthy surgical procedure involving multi-visceral resection, burdened by high morbidity due to the synergistic effect of cytoreduction, hyperthermia, and local chemotherapy cytotoxicity (9).

Over the years, CRS and HIPEC have improved the selection of more suitable patients and is now considered safe and comparable to other high-risk surgical oncology procedures in terms of complications rate (9).

In spite of this, the learning curve for CRS plus HIPEC is not standardised yet, because the surgical procedures required to clear the peritoneal metastasis are often complex and heterogenous, and the caseload is limited, even in high-volume cancer centres (10).

In our tertiary referral hospital, the surgical treatment of PC from colorectal cancer (CRC) (11) and pseudomyxoma peritonei (PMP) (12) has reached an acceptable perioperative outcome and long-term survival, with respectively a 3-year OS of 43% for CRC and a 5-year OS of 91% for PMP.

This study aimed to generate learning curves for CRS based on the performance of a single surgeon at a single institution performing cytoreductive surgery for PC from various abdominal malignancies.

## Methods

### Study design

We retrieved clinical data for 251 consecutive patients who underwent CRS for peritoneal carcinomatosis (regardless of the origin) by a single surgeon at Fondazione Policlinico Universitario A. Gemelli IRCCS between January 2016 and December 2020. All CRS were performed by a single experienced general surgeon (AD) with no previous experience in cytoreductive surgery for carcinomatosis. AD completed the European School of Peritoneal Surface Oncology (ESPO) training during the study time-period. He was mentored along the process by another experienced general surgeon (FP), who also had already experience with peritoneal surgery and HIPEC.

We analysed the learning curve for CRS using the cumulative summation (CUSUM) and the risk-adjusted cumulative summation (RA-CUSUM) methods (13). The operation time was assessed with the CUSUM method. The RA-CUSUM was calculated using a composite variable (surgical failure) that merges all the variables presumably involved into the learning process and creates a curve plotting change in the success rate over an increasing number of cases. Surgical failure was defined as the occurrence of at least one of the following events: major post-operative complications (Clavien–Dindo grade ≥3), blood loss ≥500 mL, incomplete cytoreduction. Three learning phases were thus derived from the RA-CUSUM analysis, and were compared in terms of perioperative outcomes.

All patients provided written informed consent and entered a follow-up program. Data were collected and stored in a prospectively maintained database. The study was approved by the local Institutional Review Board (IRB). We use the STROBE statement checklist (v 4.0) for our research.

### Preoperative evaluation

After a complete preoperative workup, all patients were reviewed at our institutional peritoneal disease multidisciplinary team (MDT). The extent of peritoneal disease was subsequently assessed by diagnostic laparoscopy, and the peritoneal carcinomatosis index (PCI) scoring system was recorded for each patient. The decision of the multidisciplinary team in offering cytoreductive surgery depends on the primary oncological disease:

- *Pseudomixoma Peritoneii:* CRS and HIPEC were offered to all medically fit patients with PMP. The main goal was to achieve a complete cytoreduction; however, if this was not possible due to the extension of the disease, maximal tumour debulking was performed.
- *Colorectal and gastric peritoneal metastasis:* CRS and HIPEC was offered in case of potentially complete cytoreduction of peritoneal disease without extra-abdominal metastases. Patients not eligible for cytoreductive surgery were referred for systemic chemotherapy.
- *Ovarian cancer, mesothelioma, and other less common histology:* before progressing to CRS and HIPEC, these cases were discussed by the institutional MDT.

### Cytoreductive surgery with HIPEC

Following the laparotomy, a revaluation of PCI was performed. The surgical intent was to obtain a maximal cytoreduction and perform the HIPEC. Patients who could not achieve a complete cytoreduction, generally due to the extent of disease or their general conditions, underwent a maximal tumour debulking without HIPEC. Cytoreductive surgery was performed using the Sugarbaker technique (10). When the disease was limited, patients underwent selective peritonectomy. The surgical purpose was to remove all visible peritoneal metastases through diaphragmatic, parietal anterior, and pelvic peritonectomy with greater and lesser omentectomy. In addition, multiple organ resections were performed depending on disease involvement if an CC-0/1 resection could be achieved. Organ resections included segmental colectomy, proctectomy, small bowel resections, gastrectomy (partial or rarely total), segmental liver resection, cholecystectomy, splenectomy, and hysterectomy with bilateral salpingo-oophorectomy in females. The HIPEC procedure was then performed using the closed technique. In 8 cases, the HIPEC procedure was performed using CO_2_ technology (14).

HIPEC regimens varied according to histology: oxaliplatin were used for colorectal cancer, mitomycin for colorectal cancer, pseudomixoma peritoneii and gastric cancer, whereas cisplatin for gastric cancer, ovarian cancer and mesothelioma. The target temperature, likewise, was set between 40–42 °C and the time duration was 60 or 90 min. Glucose 5% solution was used for oxaliplatin and physiologic solution 0.9% for other drugs. An adequate intra-abdominal patient filling volume was 2–2.5 L/mq.

### Data

The clinical and pathological variables for each patient were retrospectively reviewed. The following clinical variables were recorded: age, sex, body mass index (BMI), Eastern Cooperative Oncology Group (ECOG) performance status (PS), primary tumour location, operative time, PCI score, completeness of cytoreduction (CC score), blood loss and post-operative complications, which were divided into minor (Clavien-Dindo I-II), and major (Clavien-Dindo ≥III3) (15).

Intraoperative blood loss was calculated, at the end of the procedure, by the combination of absorbent materials (number of used gauzes) and volume of blood in canisters. The 500 mL cut-off was choice considering previous literature data (16) and present NICE guidelines (17, 18). The completeness of the cytoreduction (CC) score was determined at the end of each procedure. CC-0 reflected no remaining visible disease. CC-1, 2, and 3 implied remaining diseases less than 2.5 mm, 2.5 to 2.5 cm, and greater than 2.5 cm. The procedures were divided into “complete cytoreduction” and “incomplete cytoreduction” based on the primary tumour histology. Colon cancer, gastric cancer, and mesothelioma CC-0 were deemed complete cytoreduction, while CC-1/2/3 cases were considered incomplete (19). CC-0/1 were considered a complete cytoreduction in PMP and mesothelioma (20, 21).

### Statistics

Descriptive statistics were used to describe the patients’ surgical and pathological characteristics. Continuous variables are reported as medians and ranges, and categorical variables are reported as numbers and percentages of the overall group.

We examined the learning curve for CRS using CUSUM and RA-CUSUM analyses. The patients were ordered chronologically. The CUSUM analysis for operation time (CUSUMOT) was defined as:

$$CUSUM_{OT}=\overset{n}{\underset{i=1}{\sum }}(x_{i}-\mu )$$

where $x_{i}$ is the single patient’s operation time, and μ is the mean operation time. The RA-CUSUM analysis was defined as

$$RACUSUM=\overset{n}{\underset{i=1}{\sum }}(x_{i}-\tau )+(-1{)}^{xi\;}P_{i}$$

where $x_{i}=1$ indicates the presence of surgical failure, otherwise, $x_{i}=0$; τ represents the event rate, and $P_{i}$ is the expected surgical failure rate derived from a logistic regression model with a backward stepwise selection of the variable procedure. The entire series is plotted from left to right on the horizontal axis. The curve moves down for each success and up for each failure. We delineated the end of the learning process as the point where the curve reached the steady state (13, 22).

Based on the trend displayed by the curve, the whole sample was divided into three phases: phase 1, “the learning phase”, phase 2, “the experienced phase”, and phase 3, “the mastership phase”. The different phases were compared using the *χ*^2^ test and Student *t*-tests for parametric estimations and Wilcoxon Mann-Whitney U test for nonparametric estimations. *p* ≤ 0.050 was considered statistically signiﬁcant. Statistical analyses were performed using SPSS version 24.0 (IBM, Armonk, New York, USA) software for Windows.

## Results

Two hundred fifty-one patients underwent CRS for peritoneal carcinosis from various origins during the study period. Patient characteristics are shown in Table 1. The median age was 59 years (range: 26–86 years). One hundred and forty-five patients were females (57.8%), and 106 were males (42.2%). Considering the primary cancer sites, 115 (45.8%) were colorectal cancer, 19 (7.6%) were gastric cancer, 48 (19.1%) were PMP, 16 (6.4%) were mesothelioma, 42 (16.7%) were ovarian cancer, and 11 were from various origin (pancreatic, biliary) and were grouped as other. The median PCI score calculated after laparotomy was 12 (range 3–30). Two hundred and twenty-three (88.8%) patients received a complete cytoreduction, while 28 (11.2%) received an incomplete cytoreduction. The median operating time was 502 (140–900) min. The complete operative details are shown in Table 2.

**Table 1:** Baseline characteristics and operative details.

Variable	*N* (%)
Gender	
Female	145 (57.8)
Male	106 (42.2)
Age, years [median (range)]	59 (26–86)
BMI [median (range)]	24.2 (17–41.7)
ECOG PS	
0	139 (55.4)
≥1	112 (44.6)
Primary tumor histotype	
Colorectal	115 (45.8)
Gastric	19 (7.6)
PMP	48 (19.1)
Mesothelioma	16 (6.4)
Ovarian	42 (16.7)
Other	11 (4.4)
Operative time, min [median (range)]	500 (140–900)
PCI [median (range)]	12 (3–30)
Surgical Procedures	
Right colectomy	91 (36.3)
Left colectomy	18 (7.2)
Rectal resection	122 (48.6)
Pelvic peritonectomy	163 (64.9)
Hysterectomy/oophorectomy	122 (48.6)
Diaphragmatic peritonectomy	134 (53.4)
Mesenteric cytoreduction	54 (21.5)
Omentectomy	181 (72.1)
Gastric resection	19 (7.6)
Small bowel resection	65 (25.9)
Segmental liver resection	6 (2.4)
Splenectomy	65 (25.9)
Ostomy	93 (37.1)
Completeness of Cytoreduction (CCR)	
Complete	223 (88.8)
Incomplete	28 (11.2)
HIPEC	
No	41 (16.3)
Yes	210 (83.7)
Blood loss >500 mL	
No	182 (72.5)
Yes	69 (27.5)

**Table 2:** Postoperative complications.

Variable	*N* (%)
Complications	
No	145 (57.8)
Yes	106 (42.2)
Complication grade (Clavien-Dindo)	
Grade I–II	64 (25.5)
Grade III–IV	42 (16.7)
Postoperative mortality	3 (1.2)
Postoperative ileus	10 (3.9)
Pulmonary complications	21 (8.4)
Postoperative bleeding	9 (3.5)
Abdominal collection	20 (7.9)
Genitourinary infection	11 (4.4)
Anastomotic leak^a^	13 (3.9)^a^
Surgical site infection	18 (7.2)
Renal failure	4 (1.6)

^a^ *Analyzed considering all the 334 anastomosis.*

All patients were managed in the intensive care unit for at least one night in the post-operative period. No major intraoperative complications occurred. There were 3 (1.2%) post-operative in-hospital deaths.

One-hundred and six (42.2%) patients developed post-operative complications: grade I/II complications occurred in 64 (25.5%) patients, and grade III/IV complications occurred in 42 (16.7%) patients. Considering major complications, 9 (3.5%) patients experience post-operative bleeding, 20 (7.9%) abdominal collections, and 13 (3.9%) anastomotic leaks (AL). Among the 9 patients with post-operative bleeding, 7 needed surgeries with relaparotomy and surgical hemostasis, while the remaining 2 patients were treated with angioembolisation.

The percentage of AL was analysed considering all the 334 anastomoses. Two (15.4%) ALs were from an ileocolic anastomosis, 1 (7.7%) from a colo-colic anastomosis and 10 (76.9%) from a colorectal anastomosis. Seven (53.8%) of the 13 patients with AL received a protective ileostomy during the first surgery. Among all the ALs, 3 of them were treated conservatively (percutaneous drainage plus broad-spectrum antibiotic therapy), while 10 patients underwent re-operation with resection of the anastomotic complex and colostomy or ileostomy, depending on the leak. The leading cause of abdominal collection was pancreatic fistula (10 patients; 3.9%). Among the 20 patients with abdominal collections, 12 were treated with percutaneous drainage plus antibiotic therapy, while the rest (8 patients) underwent relaparotomy and surgical drainage. The complete description of post-operative complications is shown in Table 2.

**Figure 1:**
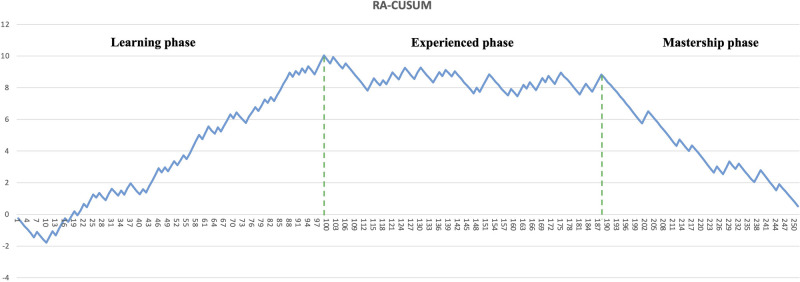
CUSUM curve of operation time (CUSUMOT).

**Figure 2:**
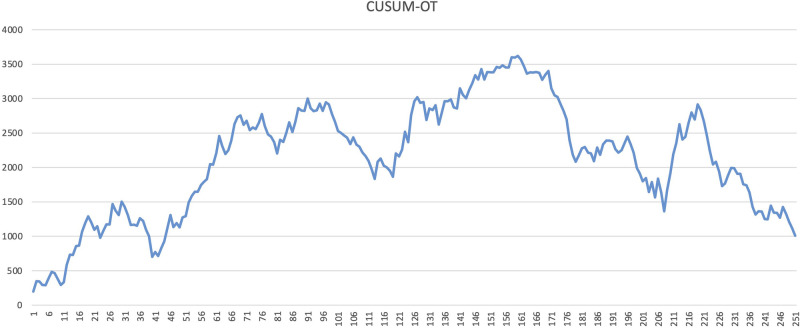
Risk-adjusted CUSUM curve (RA-CUSUM).

The learning curve for CRS was assessed using the CUSUM and RA-CUSUM methods. The CUSUM-OT curve shows an initial long plateau, with the first downward slope only after 161 cases (Figure 1). Afterwards, the reduction appears slow and inconstant, as documented by the multiple peaks and throughs in the plot (Figure 1). In the RA-CUSUM graph, the curve moved upwards for surgical failure and downwards for surgical success. The RA-CUSUM analysis revealed that the surgical failure rate was significant in the initial phase until case 99. Next, the slope remained stable until case 198 and then gradually decreased (Figure 2). Consequently, according to the RA-CUSUM curve, the CRS learning curve breaks into three different phases: phase 1, “the learning phase” (cases 1–99), phase 2 “the experienced phase” (cases 100–188), and phase 3, “the mastership phase” (cases 189–251).

Perioperative outcomes throughout the three learning phases are shown in Table 3. There were no significant differences in age, sex, BMI, ECOG grade, and primary tumour histotype among the three phases.

**Table 3:** Comparison of baseline characteristics and perioperative outcomes among the three phases defined by the RA-CUSUM analysis.

Parameters	Phase 1 (*n* = 99)	Phase 2 (*n* = 89)	Phase 3 (*n* = 63)	*p*-value	Phase 1 vs 2	Phase 2 vs 3	Phase 1 vs 3
Age (years)	55 (26–80)	60 (35–86)	61 (34–79)	0.138	0.178	0.382	0.068
Gender							
Male	39 (39.4)	45 (50.6)	22 (34.9)	0.120	0.082	0.056	0.567
BMI (Kg/m^2^)	23.9 (17–34.6)	24.7 (17–31.3)	24.6 (18–41.7)	0.080	0.469	0.101	0.031
ECOG							
≥1	47 (47.5)	36 (40.4)	29 (46)	0.605	0.333	0.493	0.858
PCI	10 (3–27)	12 (3–30)	16 (3–27)	0.001	0.096	0.029	0.001
Primary tumor histotype							
Colon	44 (44.4)	41 (46.1)	30 (47.6)	0.894	0.689	0.963	0.689
Gastric	9 (9.1)	7 (7.9)	3 (4.8)				
PMP	17 (17.2)	17 (19.1)	14 (22.2)				
Mesotelioma	5 (5.1)	7 (7.9)	4 (6.3)				
Ovarian	17 (17.2)	15 (16.9)	10 (15.9)				
Other	7 (7.1)	2 (2.2)	2 (3.2)				
Completeness of cytoreduction							
Incomplete	12 (12.1)	13 (14.6)	3 (4.8)	0.152	0.616	0.043	0.094
HIPEC							
Yes	78 (78.8)	70 (78.7)	62 (98.4)	0.001	0.561	0.001	0.001
Operative time (min)	503 (140–800)	500 (200–900)	420 (200–808)	0.031	0.963	0.017	0.021
Major complications							
Clavien-Dindo 3–5	22 (22.2)	15 (16.9)	5 (7.9)	0.019	0.230	0.085	0.013
Blood loss							
>500 mL	43 (43.4)	21 (23.6)	5 (7.9)	0.001	0.002	0.012	0.001

PCI constantly increased over the different phases and was significantly higher in phase 3 than in the other two phases (phase 3 vs phase 2: 16 vs 12, *p* = 0.029; phase 3 vs phase 1: 16 vs 10, *p* = 0.001).

Concerning the completeness of cytoreduction, even though there was no consistently significant reduction among all three phases, the rate of incomplete CCR was significantly lower in phase 3 with respect to phase 2 (4.8% vs 14.6%, *p* = 0.043). Moreover, a trend of lower incomplete CCR was observed in phase 3 than in phase 1, though not significant (4.8% vs 12.1%, *p* = 0.094). Consequently, the HIPEC administration rate increased across the three phases (*p* = 0.001) but was significantly higher in phase 3 than in the other two phases (phase 3 vs. phase 2: 98% vs. 78.8%, *p* = 0.001; phase 3 vs. phase 1: 98% vs. 78.8%, *p* = 0.001).

The rates of major post-operative complications decreased significantly over the three phases (*p* = 0.019). Despite the rates of post-operative complications not being significantly different among phases 1 and 2 and phases 2 and 3, there was a significant reduction in complication rates in phase 3 compared to phase 1 (phase 3 vs phase 1: 7.9% vs 22.2%, *p* = 0.013).

Operative time decreased significantly over the three phases (*p* = 0.031) and was significantly lower in phase 3 than in the other two phases (phase 3 vs phase 2: 420 min vs 500 min, *p* = 0.017; phase 3 vs phase 1: 420 min vs 503 min, *p* = 0.021). Blood loss constantly and significantly decreased among the three phases (*p* = 0.001).

## Discussion

Considering the results of the case series published in the literature, the number of operations necessary to overcome the learning curve varies from 130 to 220 cases (23–26). The first report was in 2007 when Smeenk et al. (23) showed a significant increase in complete cytoreduction from 35.6% to 65.1% after 130 cases. A similar learning curve of 140 cases based on completeness of cytoreduction and severe morbidity was reported by Kusamura et al. in 2012 (24). In two other series, the operative outcomes are improved following 220 (25) and 180 (26) cases, respectively.

In our understanding, such heterogeneity could be due to the use of different analytical methods and outcomes to evaluate surgical proficiency in CRS. In other complex surgical procedures, such as pancreaticoduodenectomy (22), the operation time is frequently used as a primary outcome to measure the surgical skill improvement and it decreases with the surgeons’ experience. However, especially in CRS, operation time is affected by several factors, such as PCI, location of PM, primary disease, regardless of the surgeon's skill and experience. In addition, the relationship between operative time and post-operative complications is not well defined yet. Furthermore, CUSUMOT plot shows a rather steady trend in our paper, and only after 161 cases a late and slow reduction is visible. Additionally, several inconstant peaks were visible in the late period after the 161 cases (Figure 1), suggesting an inconsistent reduction in the operative time.

To overcome this limitation, we outline a composite variable (surgical failure) that merges all the parameters supposedly entailing the learning process.

Among the three phases defined by the RA-CUSUM curve, we found no difference in the rate of incomplete cytoreduction, but there was a significant reduction in the rate of severe postoperative complications.

During the first 99 cases, we found a complication rate of 22.2%, comparable with the one reported in the literature (27). Moreover, this value is higher than 16.7%, which is the median value of the major complication rate of our whole series (Table 3). Furthermore, the rate of incomplete cytoreduction in this phase is 12%, comparable with the value reported by other papers (24, 27, 28).

This means that our results are within acceptable limits (i.e. minimal level of proficiency) in terms of morbidity, mortality, and completeness of cytoreduction, also during the initial phase, when surgeries are performed without having gained the highest level of expertise.

After the 99th case, the RA-CUSUM curve reaches a steady-state until the 188^th^ case (i.e. the experienced phase). A possible explanation for this “deadlock period” without any apparent improvement in surgical skills could be that we implemented our surgical indications after gaining experience and autonomy, including patients with a higher disease burden. As a matter of fact, in our series, the PCI and the consequent surgical challenge gradually increases during the study period (Table 3). Hence, the improved skills acquired in this phase were partially hidden by the significant surgical abilities required to manage a more significant disease load. Moreover, the results obtained in this phase (major complication rate 16.9%, incomplete cytoreduction rate of 14.6%) are acceptable and widely comparable with other data reported from other series (27, 29). Hence, a relatively long period is necessary to improve these results considering the advanced technical skills needed to face a more extensive disease.

Following a relatively long period of stability, the RA-CUSUM slope takes a gradual decrease after the 189th case (i.e. the mastership phase), with a clear improvement of all the surgical outcomes. In this phase, the achieved surgical abilities allow dealing with a more extensive and challenging disease (median PCI = 16), achieving a remarkably low rate of incomplete cytoreduction (4.8%) and complication rate (7.9%). Moreover, our major complication rate is lower than other series reported in the literature (27–31). The improvement of the rate of incomplete cytoreduction accounts for the significantly higher rate of HIPEC administration in the last phase compared with the other two (Table 3).

Additionally, to highlight further the surgical proficiency acquired in the mastership phase, we found a significant reduction in operative time compared with the learning phase (*p* = 0.021) and the experienced phase (*p* = 0.017).

Pondering on our results, the achievement of the technical proficiency required 189 cases, which is comparable with the 130–220 cases reported in the literature. Before achieving the complete proficiency and after the first 99 cases, the surgeon could safely perform CRS in patients with a moderate extension of the disease, obtaining a complication rate and an incomplete cytoreduction rate widely comparable to other series in the literature.

In this study of an experienced surgeon without prior experience of CRS and HIPEC, the incidence of major post-operative complications and the rate of incomplete cytoreduction, even in phase 1, were comparable with the incidence in prior studies (24, 27, 28). The reason for these results could be the accurate and targeted selection of patients with a low tumour burden in the early phase, performed under the direct supervision of the mentor and in a multidisciplinary context. In this way, the surgeon could deal with the type of disease most suited to his surgical skills at every stage of his learning process. Therefore, the mentorship by another experienced general surgeon with previous experience with peritoneal surgery and HIPEC and a well-defined multidisciplinary team is crucial to expedite an otherwise long and steep learning curve, decrease its untoward outcomes in the early period, and ameliorate oncological outcomes.

As shown in this study and earlier studies, the long and insidious learning curve before achieving surgical proficiency is the main obstacle to the safe diffusion of CRS and HIPEC. Estimating that the average amount of CRS and HIPEC at reference high-volume centres varies from 24 to 123 cases per year, achieving technical competency can take several years (32).

This study has some limitations. First, our analyses did not investigate whether the initial training period has some impact on oncological outcomes. However, the incomplete cytoreduction rate, which represents a surrogate marker for oncologic outcomes, was not inferior to the historical data from previous reports (27–31). And secondly, it is unclear which technical aspect of CRS or HIPEC provided the most outstanding contribution to the long learning curve. Identifying the crucial steps in the learning process could shorten the achievement of surgical proficiency.

In conclusion, a long learning curve was necessary even for an experienced general surgeon to achieve technical proficiency. Moreover, the technical aspect of CRS is relatively unfamiliar to most general surgeons. Hence, a mentorship model in high-volume centres by surgeons with experience and knowledge of this disease should be paramount to reduce the learning curve. Therefore, considering a possible future direction, it is crucial to develop standardised training programs to shorten the learning process reduce morbidity and mortality, and improve oncologic outcomes.

## Data Availability

The raw data supporting the conclusions of this article will be made available by the authors, without undue reservation.
